# Demographic features, health status, and behavioral changes associated with cannabidiol use in the Dog Aging Project

**DOI:** 10.3389/fvets.2025.1666663

**Published:** 2025-11-28

**Authors:** Kendra D. Conrow, Richard S. Haney, Michael H. Malek-Ahmadi, Julia D. Albright, Barbara L. F. Kaplan, Noah Snyder-Mackler, Kathleen F. Kerr, Yi Su, Daniel E. L. Promislow, Emily E. Bray, Dog Aging Project Consortium, Audrey Ruple, Brooke Benton, Benjamin S. Wilfond, Erica C. Jonlin, Elhanan Borenstein, Elinor K. Karlsson, Evan L. MacLean, Annette L. Fitzpatrick, Joshua M. Akey, Jessica M. Hoffman, Jing Ma, Matt Kaeberlein, Kathleen F. Kerr, Kate E. Creevy, M. Katherine Tolbert, Matthew D. Dunbar, Amanda E. Coleman, Marta G. Castelhano, Noah Snyder-Mackler, Daniel E. L. Promislow, Sandi Shrager, Stephen M. Schwartz, Silvan R. Urfer, Virginia R. Fajt, Maxwell C. K. Leung

**Affiliations:** 1 School of Mathematical and Natural Sciences, Arizona State University, Glendale, AZ, United States; 2 Cannabis Analytics, Safety and Health Initiative, Arizona State University, Glendale, AZ, United States; 3 Banner Alzheimer's Institute, Phoenix, AZ, United States; 4 College of Veterinary Medicine, University of Tennessee, Knoxville, TN, United States; 5 College of Veterinary Medicine, Mississippi State University, Mississippi State, MS, United States; 6 School of Life Sciences, Arizona State University, Tempe, AZ, United States; 7 School of Public Health, University of Washington, Seattle, WA, United States; 8 Jean Mayer USDA Human Nutrition Research Center on Aging, Tufts University, Boston, MA, United States; 9 College of Veterinary Medicine, University of Arizona, Tucson, AZ, United States

**Keywords:** canine (dog), behavior, supplement, human animal bond, cannabidiol

## Abstract

**Introduction:**

In recent years, there has been an increase in access to and interest in cannabidiol (CBD) and hemp products, including use in companion dog supplements.

**Methods:**

Using survey data from the Dog Aging Project (DAP), we characterized how human decisions around administering CBD and hemp products are informed by owner and dog demographics, the legal status of medical cannabis in their state, and the dog’s health status. Furthermore, in a subset of longitudinal data, we investigated if frequent CBD use over time was associated with behavioral changes in the dogs. The patterns of canine CBD use were first categorized by sorting owner responses into frequent use, infrequent use, and no use. They were then analyzed for associations with location and general demographic features. Further cross-sectional analyses identified the association of CBD use with different medical conditions, as well as age-associated behavioral changes in dogs with consistent CBD use for at least 2 years as compared to dogs with no use.

**Results:**

Cannabidiol use tended to be higher in states with legalized medical cannabis. Dogs with dementia, osteoarthritis, cancer, epilepsy, hip dysplasia, clinical sign–based gastrointestinal disorders, and chronic diarrhea were more likely to use CBD as compared to dogs without those conditions. Dogs with consistent CBD use showed a decline in the intensity of aggressive behaviors over time compared to a more steady trend among non-use dogs.

**Discussion:**

The use of CBD in dogs is linked to owner perceptions of what CBD could treat or alleviate. The decline in the intensity of aggressive behaviors suggests the potential use of CBD in dog training. Further studies are needed to advance our understanding of this novel supplement at the mechanistic, clinical, and population levels. This will benefit the health of humans and dogs alike.

## 1 Introduction

The production and sale of *Cannabis sativa* (cannabis and hemp) in the U. S. have been largely prohibited by the Controlled Substance Act since 1970 (1). This changed in 2018 when the passage of the Farm Bill legalized the production and sale of hemp and hemp-derived products (2), such as cannabidiol (CBD). The prevalence of these products has since risen considerably in the human consumer market (3). The popularity of these products has also increased in companion animals for a variety of health- and behavior-related reasons, which are often influenced by the animal owners’ perceptions of the health benefits of such products (4). This interaction between humans and companion animals is part of extended ultrasociality and the evolutionary development of human and animal bonds (5).

Cannabidiol, a non-psychedelic component of *Cannabis sativa*, has been hypothesized to have therapeutic effects for a wide range of conditions in humans (6). The most well-established use is in the treatment of seizures (7). A CBD product extracted from the cannabis plant is FDA-approved to treat seizures associated with Lennox–Gastaut syndrome, Dravet syndrome, and tuberous sclerosis complex (7). Synthetic CBD has been approved in humans to treat nausea and vomiting related to chemotherapy treatment. Cannabidiol has also been shown to have anti-inflammatory properties (8) and is commonly used to treat chronic pain (9). Another popular use for CBD is as a therapy for mental health conditions like anxiety and poor sleep by acting on neurotransmitter systems, such as serotonin and gamma-aminobutyric acid (10). While the possible health benefits and risks of CBD and hemp products in humans are promising and have been active areas of research, current clinical findings are often limited by sample size and study duration (11, 12).

Living together in the same environment with humans, companion dogs can provide a useful model for evaluating the potential health and behavioral effects of CBD and hemp products. Currently, the effects of CBD and hemp have only been evaluated in a small number of canine studies of healthy and clinical ill dogs to date (13–15). Owner reports and veterinary assessments have both shown benefits in reducing pain associated with osteoarthritis (16, 17), pruritus from environmental allergies (18, 19), and some measurements of anxiety in dogs (20, 21). Cannabidiol appears to be safe in these studies and has been evaluated for up to 6 months (22), but the long-term safety and efficacy of CBD products is largely unknown.

The Dog Aging Project (DAP) is a multi-year community science project that has reached over 50,000 dog owners and may be able to provide valuable longitudinal data for CBD use in companion dogs (23, 24). We gather reports about participating dogs’ experiences and demographic details over the lifespan of the dogs in an environment where humans and dogs live together. The overall goal of the DAP is to understand how the complex interactions of diet, lifestyle, and the environment can influence health and aging. This One Health approach aims to generate knowledge that could benefit humans and dogs alike.

In this study, we used the DAP survey data to characterize the demographic features, health status, and behavioral changes with CBD and hemp use in companion dogs throughout the U. S. Our objectives were to (i) determine how the dog and owner demographic features are associated with the owners’ decision to use CBD and hemp; (ii) characterize the cross-sectional associations between health conditions and CBD and hemp usage, which could reflect dog owner perceptions of their applications for health; and (iii) identify the age-associated behavioral changes of dogs with long-term CBD and hemp use. To the authors’ knowledge, this represents the largest comprehensive assessment of CBD and hemp use for companion animals to date.

## 2 Methods

### 2.1 Study population and recruitment

The Dog Aging Project is a community science project that aims to understand how genes, diet, lifestyle, and the environment influence aging and health outcomes in companion dogs (23, 24). Recruitment of owners in the DAP generally relies on various forms of online media or word of mouth and is ongoing with more participants every year (23). We used the DAP 2023 Curated Data Release version 1.0 (released on 9 February 2024) containing dog data from 47,444 owners that included the baseline Health and Life Experiences Survey (HLES) and the Annual Follow Up Surveys (AFUS) between 26 December 2019, and 31 December 2023 (25). Both surveys consist of multiple sections that collect information about dog demographic characteristics, physical activity, environment, dog behavior, diet, medications and preventatives, health status, and owner demographic characteristics. These sections are publicly available on GitHub (26). Data were collected and managed using REDCap (Research Electronic Data Capture) electronic data capture tools hosted through the DAP (27, 28).

### 2.2 Dog Aging Project survey data

Dogs were enrolled in the DAP after the baseline HLES was completed by the dog owners. The AFUS is given every year afterwards. The sections of HLES and AFUS used for cross-sectional and age-associated analyses included “dog demographics,” “physical activity,” “environment,” “dog behavior,” “diet,” “medications and preventatives,” “health status,” “owner contact,” and “owner demographics.” In the cross-sectional analysis, we used the most recently completed survey data of demographics and health conditions. For age-associated analysis, we used HLES and AFUS that were completed 9–15 months apart to assess the behaviors observed among dogs over time. In both analyses, we also filtered out surveys for dogs older than 18 years [which were considered outliners (29, 30)], as well as dogs with no age data. The survey variables examined in the cross-sectional and age-associated analyses are described in the following sections.

### 2.3 Cannabidiol and hemp use

The frequency of CBD and hemp use in a dog was determined using owner responses in HLES and AFUS. “Frequent use” referred to owner-reported “non-prescription medication” or “daily supplement” use of CBD and hemp products. “Infrequent use” referred to CBD and hemp supplement use less than once a day. “No reported use” referred to no report of any CBD and hemp products. In the age-associated behavioral analysis, we only compared dogs with consistent use (i.e., frequent use over two consecutive surveys or more) to dogs with no use reported in any survey. Prior to July 2022, HLES had no check-box question regarding the use of CBD and hemp products, so only the free-response sections for “daily supplements,” “infrequent supplements” and “non-prescription medications” were used to capture CBD and hemp use based on the mentions of “CBD,” “hemp,” or “cannabidiol.” The AFUS offered a selection for CBD use as a “daily supplement” or “infrequent supplement,” but not under “non-prescription medications.” The CBD supplement selection and the three free-response descriptions that mentioned “CBD,” “hemp,” or “cannabidiol” were considered as CBD and hemp use for our analysis and “CBD” will refer to both CBD and hemp use hereafter. If a survey had both infrequent and frequent use marked, it was counted as “frequent use.”

All surveys after July 2022 include a comprehensive diet section, which has CBD as a specific supplement and asks about frequency with multiple choices for the frequency at which the supplements are given. Any CBD supplement use with one or more per day selected was considered frequent to ensure all survey results were consistent with the previous classifications. All other frequencies of indicated CBD supplement use (e.g., “weekly,” “monthly,” “less than monthly,” “other”) were counted as infrequent. Only one CBD use classification was used for each survey; if any two sections differed in the indicated frequency, the survey would be considered as indicating the higher level of use (i.e., “frequent use”).

### 2.4 State legal status of medical cannabis

Cannabidiol is not considered a controlled substance in the U. S. since 2018 (1, 2). In comparison, medical cannabis was only legalized in Washington, D. C. and 38 states as of December 31, 2023 (31). To test the hypothesis that the dog owners’ perception of medical cannabis use in humans affects their use of CBD on their dogs, we compared the prevalence of CBD use in dogs in U. S. jurisdictions with no legal access to cannabis (or with legalized CBD or low THC programs only) to those with legal access to medical cannabis. The four jurisdictions that enacted laws allowing medical cannabis during the DAP survey data collection in 2020–2023 were excluded from this analysis. The legal status of cannabis in each state was obtained from the National Conference of State Legislatures website (31).

### 2.5 Health conditions

We examined two categories of health conditions that owners could be giving their dogs CBD for as treatments. The first category was inflammatory conditions such as dermatitis, osteoarthritis, thrombocytopenia, hemolytic anemia, and inflammatory bowel disease. The second category consisted of other conditions for which CBD is frequently used as symptom management in dogs and humans: cancer (32, 33), epilepsy (7, 34), and dementia (35). We also included hip dysplasia since it could lead to secondary osteoarthritis and joint pain in dogs and humans (36, 37). In addition to the beneficial effects perceived by the owners, we included potential side effects of CBD such as clinical-sign–based gastrointestinal (GI) disorders – including chronic vomiting, chronic diarrhea, constipation, fecal incontinence, and bilious vomiting syndrome as outlined by Schmid et al. (38). We identified dogs affected by these conditions at any point in their lives for the analysis described below.

### 2.6 Health-related covariates and logistic regression

In the cross-sectional analysis, we first calculated the prevalence of CBD use in dogs with the above health conditions. Next, we determined if any of these conditions had a significant association with CBD use using logistic regression as follows:

logit (P(CBD use)) = β_0_ + β_1_*Dog Age + β_2_*Spayed Female + β_3_*Intact male + β_4_*Neutered male + β_5_*Dog weight + β_6_*Owner’s annual income range + β_7_*Owner’s maximum education level + β_8_*Owner’s age range + β_9_*Presence of specified health condition.

We included dog age, dog weight, and owner age, income, and education level as covariates. Dog sex and sterilization status were also included as a single covariate with four factors: intact female, spayed female, intact male, and neutered male. Exp (β_9_) estimates the adjusted odds ratio summarizing the association between the specified health condition and CBD use. This model was run using the R Stats Package (Version 4.4.1) in RStudio (Version 2025.05.0 + 496).

### 2.7 Physical activity and cognitive training

Physical activity was calculated using a principal component analysis on three survey questions: (i) average daily time spent active reported in hours and minutes; (ii) average activity intensity level reported using a three-tier scale; and (iii) lifestyle activity level reported using a three-tier scale as described in Bray et al. (30) The principal component analysis results in a physical activity score that has a mean value of 0, with higher scores indicating more physical activity, and lower scores indicating less physical activity. Cognitive training of each dog was counted as a presence (1) vs. absence (0) variable with no distinction in levels of training. The history of cognitive training for each dog was determined based on the presence of primary and secondary activities that would necessitate cognitive training to some degree. Dogs who were determined to have a history of training included show dogs, working dogs, service dogs, and dogs participating in activities including agility training, field trials, hunting, obedience training, and search and rescue.

### 2.8 Sensory and motion impairment

The presence of sensory and motion impairment was incorporated as a covariate in the age-associated analysis. Sensory impairments included any permanent conditions that greatly impacted sight or hearing, as well as one semi-permanent condition, cataracts, which was classified as a sensory impairment if there was no indication of a corrective surgery associated with it. Health conditions classified as motion impairments included neurological and orthopedic conditions that greatly impacted a dog’s mobility for their remaining lifetime. Conditions that met these standards for impairment are listed in Supplementary Table 1.

### 2.9 Behavioral scores

Dog behaviors were assessed using a modified version of the Canine Behavioral Assessment & Research Questionnaire (C-BARQ) (39, 40) which was completed by owners annually in the “dog behavior” section of HLES and AFUS. All 42 questions were categorized into one of the three main behaviors—aggression, fear, and agitation. The aggression score was based on questions about aggression and those about chasing wild animals. Fear was based on the questions about fear and behaviors when dogs were left alone such as restlessness, scratching, and whining. Agitation as a group consisted of all responses not classified as fear or aggression. Abnormal agitation covered a subset of the agitation behaviors considered to be abnormal. Additionally, 22 of the 42 questions were classified into three groups for secondary behavioral score calculation: (i) reactions to familiar circumstances; (ii) reactions to unfamiliar circumstances; and (iii) reactions to animals. All seven behavioral scores were calculated by averaging the score of questions in each category. The response to the questions was on a scale of 1–5, with 1 indicating the least intense behaviors and 5 the most. The full list of dog behavior questions, and their classification, can be found in Supplementary Table 2.

### 2.10 Age-associated behavioral analysis

We used a linear mixed-effect model to assess dog behaviors over age, comparing dogs with consistent CBD use to dogs with no reported use. Age was used as the time variable in the model. We only included dogs with data from at least two consecutive surveys. Additionally, an approximate one-year gap must have separated all surveys to standardize response times. In addition to age, we included each dog’s sex and sterilization status, weight, physical activity score, history of cognitive training, sensory impairment, and motion impairment, as well as household characteristics (e.g., number of children, adults, other outdoor animals, and other indoor animals) as covariates in our model:


$$\begin{array}{l} \mathrm{Dog}\;{\text{behavior score}}_{i}=\beta_{0}+{\beta_{1}}^{*}\text{Spayed}\;{\text{female}}_{i}\\ +{\beta_{2}}^{*}\text{Intact}\;{\text{male}}_{i}+{\beta_{3}}^{*}\text{Neutered}\;{\text{male}}_{i}+{\beta_{4}}^{*}{\text{Weight}}_{i}\\ +{\beta_{5}}^{*}\text{Physical}\;{\text{activity score}}_{i}+{\beta_{6}}^{*}\text{History}\;{\text{of training}}_{i}\\ +{\beta_{7}}^{*}\text{Sensory}\;{\text{impairment}}_{i}+{\beta_{8}}^{*}\text{Motion}\;{\text{impairment}}_{i}\\ +{\beta_{9}}^{*}\text{Number of people}\;{\text{in household}}_{i}\\ +{\beta_{10}}^{*}\text{Number of other animals}\;{\text{in household}}_{i}+{\beta_{11}}^{*}{\mathrm{Age}}_{i}\\ +{\beta_{12}}^{*}\mathrm{CBD}\;{\mathrm{use}}_{i}+{\beta_{13}}^{*}{{\mathrm{Age}}_{i}}^{*}\mathrm{CBD}\;{\mathrm{use}}_{i}+\gamma_{\mathrm{i0}+}\;{\gamma_{\mathrm{i1}}}^{*}{\mathrm{Age}}_{i}+\varepsilon_{i}. \end{array}$$

where β_j_ are the coefficients for the fixed effects, and γ_i0_ and γ_i1_ are the random intercept and slope for each individual dog.

The model was run using the R Package nlme (R Version 4.4.1; nlme Version 3.1–164) in RStudio (Version 2025.05.0 + 496). We deem normality not a particular concern given the large sample size in this study (41). We also did diagnostics with the residual plots for dog behaviors (data not shown), which raised no concerns for homoscedasticity. We used an alpha value of 0.05 without multiple test correction. The full model output is shown in Supplementary Table 3.

### 2.11 Ethics approval statement

The University of Washington Institutional Review Board (IRB) deemed that recruitment of dog owners for the DAP and the administration and content of the HLES are human subjects research that qualifies for Category 2 exempt status (IRB ID no. 5988, effective 10/30/2018). No interactions between researchers and privately owned dogs occurred; therefore, the Institutional Animal Care and Use Committee oversight was not required.

## 3 Results

### 3.1 Demographic features of dogs with different levels of cannabidiol use

In the most recent survey for all dogs, we found 2,592 dogs (or 5.5% out of 47,355 dogs) were reported to have infrequent or frequent CBD use. The mean (SD) age of dogs with frequent use was 10.2 years (4.3) compared to 8.1 (4.1) for dogs with infrequent use and 7.6 (4.3) for dogs with no reported use. A slightly higher proportion of male dogs (5.7%) were reported to use CBD than female dogs (5.2%). The dogs with CBD use were consistently older than the ones without across all weight classes, but the sex disparity was less consistent (Supplementary Table 4). The ratios of purebred and mixed breed dogs, the weight, the sterilization status of dogs, as well as the physical activity of dogs at their most recently recorded survey were comparable across different use levels (Table 1). Owner demographics were also comparable across different use levels (Supplementary Table 5).

**Table 1:** Demographic comparison of dogs with no reported use, infrequent use, and frequent use of cannabidiol (CBD) in the Dog Aging Project at the time of the most recently completed survey (*n* = 47,355).

	No CBD use		Infrequent use		Frequent use	
	N	Percent	N	Percent	N	Percent
Total	44,767	95%	626	1%	1962	4%

Characteristic	N	Mean (SD)	N	Mean (SD)	N	Mean (SD)
Age	44,767	7.6 (4.3)	626	8.1 (4.1)	1,962	10.2 (4.3)
Weight (lbs.)	44,767	51 (30)	626	51 (29)	1,962	53 (31)
Physical activity*	35,886	−0.05 (1.0)	447	−0.04 (1.0)	1,627	−0.37 (1.0)
Aggression**	44,767	1.9 (0.5)	626	2.0 (0.5)	1,962	1.9 (0.5)
Fear**	44,766	1.9 (0.6)	626	2.0 (0.6)	1,962	2.0 (0.6)
Agitation**	44,767	2.4 (0.4)	626	2.4 (0.4)	1,962	2.3 (0.4)

	N	Percent	N	Percent	N	Percent
Female	22,282	50%	300	48%	928	47%
Male	22,485	50%	326	52%	1,034	53%
Purebred	22,577	50%	275	44%	960	49%
History of cognitive training	10,905	24%	190	30%	478	24%

*The physical activity score is based on a principal component analysis resulting in data with a mean score over all time points of 0.0 and a middle 50% of [−0.7, 0.6]. **The aggression, fear, and agitation scores were on the scale of 1 to 5, with 1 indicating the least intensity and 5 the most.

### 3.2 Prevalence of cannabidiol use in dogs and legal status of medical cannabis by states

**Figure 1:**
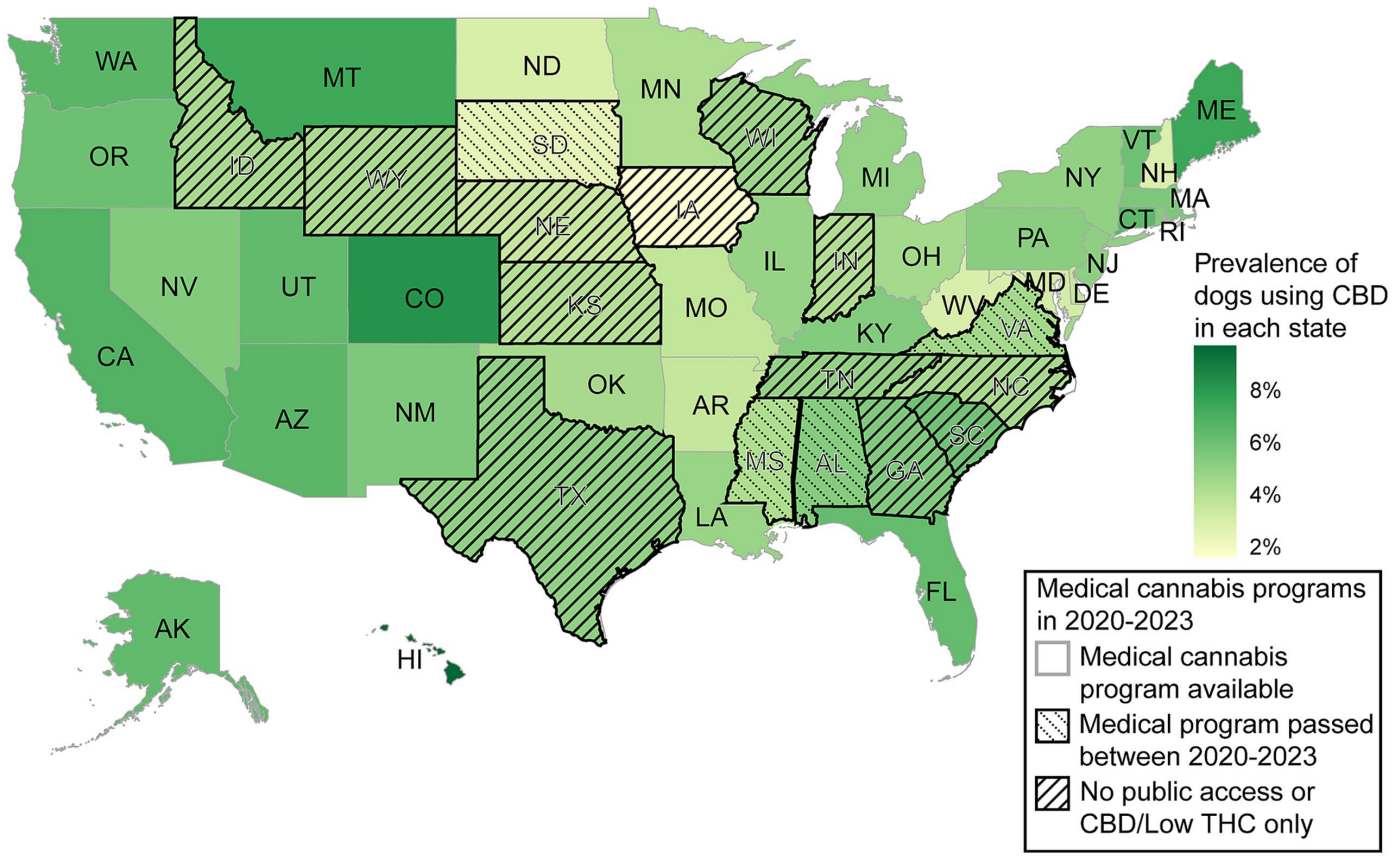
Prevalence of dogs in the 50 U. S. states and Washington, DC with reported cannabidiol (CBD) use in the Dog Aging Project (DAP) alongside the legal access of state residents to medical cannabis (according to the National Conference of State Legislatures). On average, 2,592 out of all 47,355 dogs in the DAP were reported with CBD use.

For dogs in our dataset, the prevalence of CBD use in the 50 U. S. states and Washington, D. C. ranged from 1.6 to 9.7%, with Iowa having the lowest percentage and Hawaii having the highest (Figure 1). A total of 10,808 dogs lived in 12 jurisdictions where state residents had no legal access to cannabis or could only legally obtain CBD oil. 4.7 percent of the dogs in those jurisdictions were given CBD. In comparison, 34,404 dogs lived in 35 jurisdictions where state residents had legal access to medical cannabis before the beginning of the DAP surveys. 5.8 percent of the dogs in those jurisdictions were given CBD.

### 3.3 Prevalence of cannabidiol use in different dog breeds

Combining the survey data of all dogs in every year, we found that 3,470 out of the 47,355 dogs, or 7.3%, were given CBD at any point. Of those, 2,759 reported frequent use. The prevalence of CBD use in each breed (if purebred) or primary breed (if mixed breed) were calculated based on any reported infrequent and frequent use throughout the surveys. The top 25 most common dog breeds in the DAP had a prevalence of CBD use ranging from 2.1 to 6.7% (Table 2). The highest CBD prevalence of use in breeds with >50 dogs surveyed was the purebred Leonberger at 10.3%, while purebred Scottish terrier, mixed black and tan coonhound, and mixed standard schnauzer had no reported CBD use (Supplementary Table 6).

**Table 2:** Showing the 25 most common pure and mixed dog breeds in the Dog Aging Project by prevalence of cannabidiol (CBD) use.

Breed/Primary breed	Breed status	# of dogs with CBD use	Total # of dogs	Prevalence of use
Pembroke Welsh Corgi	Purebred	24	357	6.7%
Dachshund	Purebred	34	580	5.9%
Australian Cattle Dog	Mixed	40	728	5.5%
Beagle	Mixed	32	601	5.3%
American Pitbull Terrier	Mixed	69	1,297	5.3%
Australian Shepherd	Mixed	32	631	5.1%
Siberian Husky	Mixed	25	518	4.8%
American Staffordshire Terrier	Mixed	36	762	4.7%
Chihuahua	Mixed	54	1,221	4.4%
Labrador Retriever	Mixed	137	3,149	4.4%
Australian Shepherd	Purebred	35	844	4.1%
German Shepherd Dog	Mixed	48	1,178	4.1%
Golden Retriever	Mixed	43	1,075	4.0%
Dachshund	Mixed	14	356	3.9%
Border Collie	Mixed	38	968	3.9%
Labrador Retriever	Purebred	104	2,722	3.8%
German Shepherd Dog	Purebred	39	1,129	3.5%
Chihuahua	Purebred	12	355	3.4%
Border Collie	Purebred	18	553	3.3%
Golden Retriever	Purebred	77	2,470	3.1%
Jack Russell Terrier	Mixed	11	370	3.0%
Poodle	Mixed	36	1,228	2.9%
Shih Tzu	Mixed	9	355	2.5%
Boxer	Mixed	12	479	2.5%
Poodle	Purebred	17	821	2.1%

The mixed breed designation refers to dogs that are not purebred. The first column indicates either the primary breed the owners submitted in the survey for mixed breed dogs, or the only breed for purebred dogs.

### 3.4 Dogs with certain health conditions have higher prevalence of cannabidiol use

The highest prevalence of CBD use was found to be associated with dogs with dementia at 18.2% (Table 3). The health condition with the highest number of dogs given CBD was osteoarthritis (12.5%), followed by cancer (10.0%). Of the 199 dogs using CBD that reported clinical-sign–based GI disorders, 54 reported chronic vomiting and 132 reported chronic diarrhea. After controlling for covariates, there were seven health conditions significantly associated with CBD use: dementia, osteoarthritis, epilepsy/seizures, hip dysplasia, cancer, chronic diarrhea, and clinical sign–based GI disorders (*p* < 0.05) (Table 3). There were only a small number of dogs (*n* < 60) affected with hemolytic anemia, thrombocytopenia, and clinical sign–based GI disorders other than chronic vomiting and diarrhea in our dataset, so the analysis for those conditions were excluded.

**Table 3:** Cross-sectional analysis of cannabidiol (CBD) use by dogs reporting inflammatory and other common conditions.

Health condition	# of dogs with CBD use	Total # of dogs	Prevalence of use	Adjusted odds ratio	95% CI	*p-*value
Dementia	79	433	18.2%	1.98	(1.54, 2.56)	< 0.001
Osteoarthritis	479	3,820	12.5%	1.75	(1.56, 1.96)	< 0.001
Seizures/epilepsy	125	1,174	10.6%	1.61	(1.33, 1.95)	<0.001
Cancer	437	4,384	10.0%	1.41	(1.26, 1.58)	< 0.001
Hip dysplasia	129	1,227	10.5%	1.62	(1.34, 1.96)	< 0.001
Irritable bowel syndrome	59	788	7.5%	1.17	(0.89, 1.53)	0.26
Chronic vomiting	54	769	7.0%	1.21	(0.92, 1.61)	0.18
Clinical sign–based gastrointestinal disorders	199	2,894	6.9%	1.16	(1.00, 1.35)	0.049
Chronic diarrhea	132	1,912	6.9%	1.21	(1.01, 1.46)	0.04
Dermatitis	98	1,554	6.3%	1.03	(0.84, 1.27)	0.77

Dogs with dementia showed the highest prevalence of use at 18.2% and dogs with dermatitis the lowest at 6.3%. Adjusted odds ratios compare the odds of CBD and hemp use among dogs with the condition to dogs without the condition, adjusting for dog age, sex, sterilization status, and weight, and owner age, income, and education level. Dementia, osteoarthritis, seizures and epilepsy, cancer, hip dysplasia, clinical sign–based gastrointestinal disorders, and chronic diarrhea show significant association with CBD use (*p* < 0.01), with adjusted odds ratios indicating a 98, 75, 61, 41, 62, 16, and 21% higher odds of use, respectively.

### 3.5 Consistent use of cannabidiol associated with less intense aggressive behaviors

**Figure 2:**
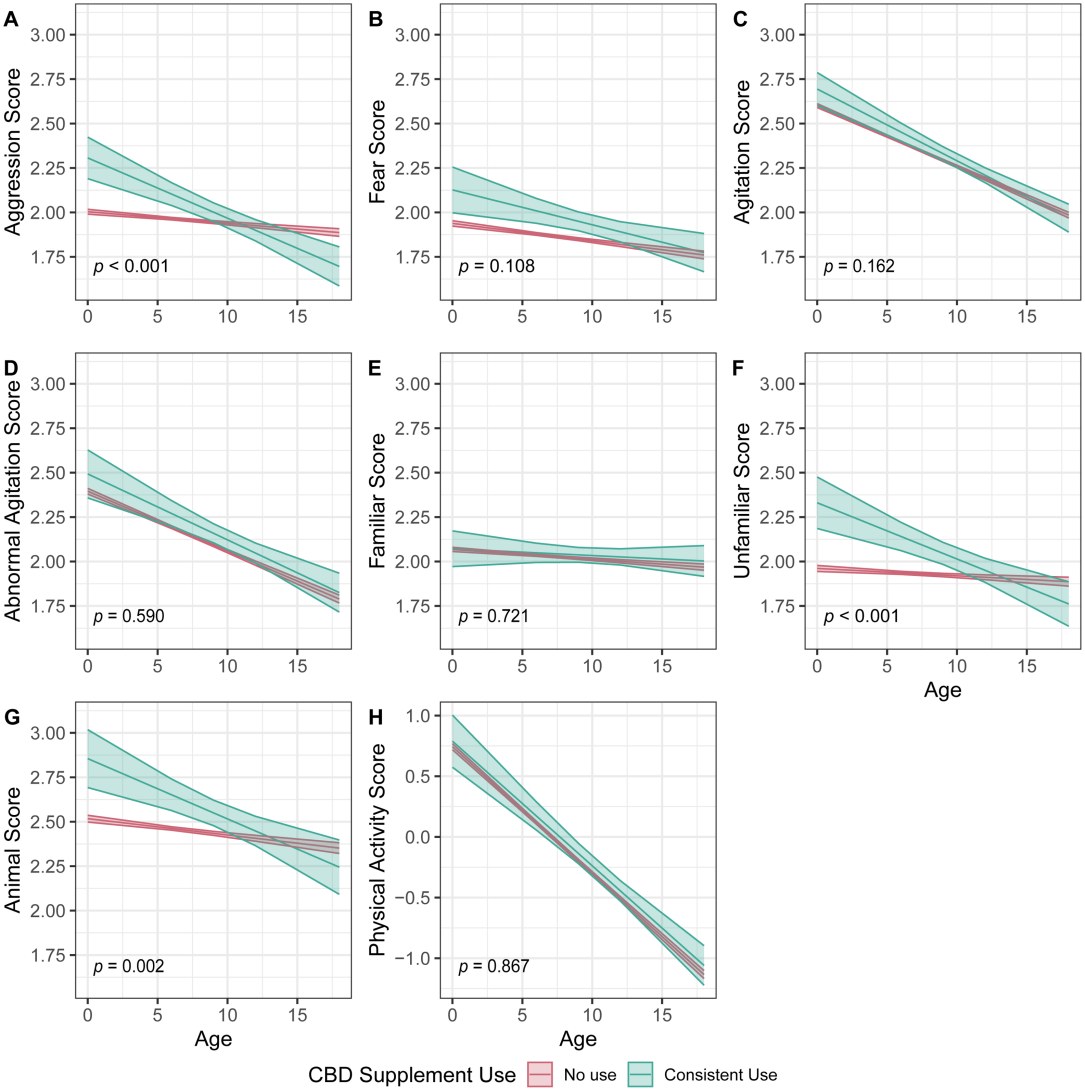
Linear mixed-effect model comparing dogs with reported consistent cannabidiol use for at least 2 years to dogs with no reported use and the change over age in dog behaviors and physical activity as reported in the Dog Aging Project (DAP). Dog behavior scores **(A–G)** are averages of select questions from the DAP. A score of 1 indicates the lowest intensity behaviors and a score of 5 indicates the most intense behaviors. Physical activity scores **(H)** are based on a principal component analysis with a standardized z-score and a mean value of 0. Higher scores indicate more physical activity and lower scores indicate less physical activity. The *p* values correspond to the interaction between age and CBD use. Each dog behavior and physical activity are treated as separate analyses.

A total of 17,730 dogs had either consistent use (*n* = 331) or consistent non-use (*n* = 17,399) of CBD for at least 2 years and were included in age-associated analyses of seven behavioral and physical activity scores (Figure 2 and Supplementary Table 3). Significant interactions were found for CBD use with age in aggression (Figure 2A), reactions to unfamiliar circumstances (Figure 2F), and reactions to animals (Figure 2G). Dogs with CBD use were found to have differing behaviors in these categories at the outset, and the intensity of their behaviors decreased at a faster rate over time as compared to dogs with no use. No significant interactions were found for CBD use with age in the other four behaviors and physical activity.

## 4 Discussion

In this study, we used the DAP’s survey data of 47,355 dogs to characterize the factors that contribute to a human dog owner’s decision to administer CBD to their dogs in the U. S. Specifically, we looked at demographic features pertaining to humans (i.e., legal access to medical cannabis by state), demographic features pertaining to dogs (i.e., age, sex, weight, and breed), and the health condition of dogs. Additionally, we compared behavioral changes over time in dogs who were consistently administered CBD to those with no reported use. Cannabidiol use was found to be more common in older dogs, aligning with trends in increased supplement use in aging. The decision to administer CBD in dogs appeared to be driven by human attitudes toward potential health benefits of medical cannabis, reflecting the role of broader societal beliefs in cannabis and cannabinoid use. Age-associated behavioral analysis suggested that dogs receiving CBD exhibited less intense aggressive behavior over time, pointing to potential long-term benefits on temperament. These findings provide a foundation for future research of CBD’s effects on the health and behaviors of animals and humans.

Our demographic analysis revealed several notable trends in CBD use in dogs. First, dogs receiving CBD tended to be older, with an average age difference of approximately 3 years compared to those not receiving CBD. This suggests that CBD may be more commonly given to senior dogs to manage age-related conditions such as arthritis (42) or dementia (43). Similar trends have also been observed in humans (44, 45). Second, there was a sex disparity, with a 9% increase in the prevalence of CBD use in male dogs as compared to females. This is different from an earlier study showing that dog sex did not influence owners’ decisions to use joint supplements in dogs (42). The reasons behind the sex difference in CBD use rates remain unclear. It is also unclear whether the CBD use rates were associated with specific chronic conditions in different breeds. Further studies should explore whether owner perceptions of behavioral differences between sexes and breeds are driving their decision to give CBD to their dogs.

A higher prevalence of canine CBD use was reported in states with more progressive cannabis legalization, suggesting that public perception and accessibility influence pet owners’ willingness to explore these products for their dogs. While the current study did not track owner use, past research has found that people who used medical and recreational cannabis had a higher rate of CBD use as compared to cannabis non-users (46). This pattern may be consistent across companion animal owners, as cat owners were more comfortable with CBD cat supplement use if they had previous experience with both prescription psychoactive and non-prescription products compared to those with no experience with either product category (47). This highlights how the cultural acceptance of supplement products may extend to their use in companion animals, driven by the belief in their potential benefits for health and well-being (48). As emerging supplements like CBD products are introduced to animals, this underscores the importance of further research to ensure safe and informed use.

Our findings revealed distinct health conditions associated with CBD use in dogs, highlighting some of the public’s perceptions of potential therapeutic uses of medical cannabis and cannabinoids. Seven health conditions—dementia, osteoarthritis, seizures/epilepsy, cancer, hip dysplasia, chronic diarrhea, and clinical sign-based GI disorders—were more commonly observed in dogs receiving CBD compared to healthy counterparts when controlling for the dog’s age, sex and sterilization status, and weight as well as the owner’s age, income, and education level. This association aligns with existing evidence supporting CBD’s efficacy for certain conditions like epilepsy (34) and inflammatory diseases such as osteoarthritis (49, 50). For other conditions, such as anxiety, dementia, cancer, and hip dysplasia, the increased use of CBD may reflect public perceptions of their use as functional food with health benefits (51, 52). Gastrointestinal disorders including diarrhea are adverse effects of oily supplements like omega-3 fatty acids (53) and CBD in dogs (13, 15, 54). Further studies are essential to evaluate whether CBD provides measurable benefits or poses potential risks.

Among the seven dog behaviors and physical activity examined in the age-associated analysis, aggression stood out as the main behavior with a statistically significant difference over age when compared to dogs not receiving CBD. Interestingly, dogs given CBD initially exhibited higher-than-average aggression levels as compared to the dogs with no CBD use. The same pattern was also seen in the secondary behavioral scores for reactions to unfamiliar circumstances and reaction to other animals. This may indicate that owners’ decisions to administer CBD was in part a reaction to their dogs exhibiting aggression. Over time, these dogs were reported to have below-average aggression eventually as compared to dogs not on CBD. The reduction in the intensity of aggressive behaviors could be the direct effect of long-term CBD use. This is consistent with the behavioral improvement observed in two earlier placebo-controlled studies of CBD use in shelter dogs and stressed dogs during separation and car travel (20, 55). However, the reduced intensity of aggressive behaviors in the current study can also be caused by other confounding factors. These include training or other medications that owners may have concurrently implemented in an attempt to reduce their dog’s aggression. Further randomized clinical trials should examine CBD’s impact on the underlying issues of aggression (e.g., inflammation, chronic pain, or other health conditions), as well as its potential role in long-term behavior management.

This study has several limitations. First, this study is based on owner-reported data. It lacks quantifiable data for the owner-reported health conditions and is subjected to the owners’ bias. Also, dogs could be mischaracterized into frequent and/or non-use if owners were misreporting and/or failing to report use. Second, the observational nature of the current study means there can be no causal inferences regarding CBD use and the observed outcomes in dogs. Rather, the current study provides additional lines of evidence to re-evaluate the positive (20, 55) and inconclusive findings (56, 57) of previous controlled studies with shorter study durations and small sampling sizes. Third, human owners in the DAP tend to be older and wealthier than the U. S. general population with a median age range of 55 to 64 and income of $100,000–$119,999, compared to the national median age of 39 and income of $80,610 (Supplementary Table 5) (58, 59). This constrains our ability to generalize these findings to low-income populations or populations with diverse economic backgrounds. This limitation might also mask potential disparities in access to veterinary care that could change the use of CBD for dogs across different socioeconomic situations. Lastly, our study lacked information on composition, cannabinoid dosages, formulations, administration routes, and product sources, making it impossible to establish the health effects and optimal usage of CBD.

## 5 Conclusion

The access to CBD products for companion dogs is comparable across the U. S., but medical cannabis for humans is only legalized in Washington, D. C. and certain states. Given this unique regulatory environment in the U. S., we explore the connection between dog owners’ perception and their decision to use CBD for canine health and behavioral benefits. The perception of CBD as a healthy supplement in humans seems to mirror the reasons that humans will administer it to their companion animals. Additionally, health conditions considered by the general public to be remediable with cannabinoid use in humans—such as cancer, epilepsy, and dementia—are also associated with increased use of CBD in dogs. The long-term use of CBD is associated with less intense aggressive behaviors in dogs. At a population level, further observational studies are needed to address the product composition, dosing regimens, formulation effects, and population-specific effects of cannabinoid-based supplements to companion dogs nationwide. At a clinical level, further targeted placebo-controlled trials are needed to evaluate CBD effectiveness in dog training and in managing behavioral and psychiatric issues. Quantifiable data such as serum cortisol levels and physical activity measurements would be crucial for future studies to characterize long term effects and better establish proper dosage for behavioral effects. These studies will advance our understanding of this novel supplement at a mechanistic level and benefit the health of humans and dogs alike.

## Data Availability

Publicly available datasets were analyzed in this study. This data can be found at: https://data.dogagingproject.org.

## DAP consortium

Audrey Ruple; Brooke Benton; Benjamin S. Wilfond; Erica C. Jonlin; Elhanan Borenstein; Elinor K. Karlsson; Evan L. MacLean; Annette L. Fitzpatrick; Joshua M. Akey; Jessica M. Hoffman; Jing Ma; Matt Kaeberlein; Kathleen F. Kerr; Kate E. Creevy; M. Katherine Tolbert; Matthew D. Dunbar; Amanda E. Coleman; Marta G. Castelhano; Noah Snyder-Mackler; Daniel E. L. Promislow; Sandi Shrager; Stephen M. Schwartz; Silvan R. Urfer; and Virginia R. Fajt.
